# Routine Medical Check-Up and Self-Treatment Practices among Community-Dwelling Living in a Mountainous Area of Northern Vietnam

**DOI:** 10.1155/2021/8734615

**Published:** 2021-04-22

**Authors:** Tam T. Ngo, Phong N. Hoang, Ha V. Pham, Dua N. Nguyen, Hoai T. T. Bui, Anh T. Nguyen, Thinh D. Do, Ngan T. Dang, Huy Q. Dinh, Dao Q. Truong, Tuan A. Le

**Affiliations:** ^1^Faculty of Health Sciences, Thang Long University, Hanoi 100000, Vietnam; ^2^E Hospital, Hanoi 100000, Vietnam; ^3^St. Paul's Hospital, Hanoi 100000, Vietnam; ^4^VNU University of Medicine and Pharmacy, Vietnam National University, Hanoi, Hanoi 100000, Vietnam; ^5^Hospital of Vietnam National University, Hanoi 100000, Vietnam

## Abstract

This study was conducted to evaluate the routine medical check-up and self-treatment behaviors of people living in a remote and mountainous setting in Northern Vietnam and identify their associations. A cross-sectional study was conducted on 175 people in August 2018 in Cao Son commune, Da Bac district, Hoa Binh. Information regarding routine medical check-ups and self-treatment behaviors was collected by using a structured questionnaire. Multivariate logistic regression was used to examine the associations. Results show that 24% of the mountainous people had routine medical check-ups in the last 12 months. The rate of self-treatment in the past three months was 33.7%. The number of chronic diseases (OR = 1.5, 95% CI = 1.0‐2.3), health information sources from radio/television (OR = 3.3, 95% CI = 1.2‐9.5), or social media (OR = 24.8, 95% CI = 1.2‐512.4) was related to routine medical check-up. People who did not have routine medical check-up were more likely to have self-treatment practice (OR = 6.3, 95% CI = 1.9‐21.1) than those who had a regular health check. Promoting health education and communication through mass media to raise people's awareness about regular health check-ups is a promising way to improve people's self-treatment status.

## 1. Introduction

Ensuring good health for all residents regardless of their geographical locations and socioeconomic status is an important goal among 17 Sustainable Development Goals [1]. However, there are still major disparities in health conditions, health care service access, and use between residents living in remote/rural and urban areas in both developed and developing countries [2–4]. Geographical distance, constrained financial resources, and insufficient health services provided by qualified health professionals are the main barriers for people in remote areas to access health care, which may also increase the risk of self-treatment and self-medication in this population [5, 6].

Self-treatment is recognized as one of the global public health issues [7]. Global research shows that the rate of self-treatment, irrespective of whether developed or developing countries, is high. For example, the rate of self-treatment is 50.0% in India [8], 50.2% in Ethiopia [9], or 35.2% in rural China [6]. In the United States, 1%-66% of residents used antibiotics without prescription [10]. Self-treatment or self-medication is defined as individuals purchasing or using drugs without a prescription or guidance of a physician or other qualified people [11]. Some conventional medicines can be used without a doctor's prescription. However, most drugs need to be strictly controlled in terms of their use, especially antibiotics [12, 13]. The benefits of self-medication or self-treatment include a reduction in the cost of medical services, convenience, and affordability, especially for people with mild symptoms. However, self-treatment can have serious consequences, such as the improper use of drugs, uncontrolled side effects, and an increased risk of drug resistance [14, 15]. Also, self-treatment may delay health facility visits to detect actual illness [15].

Routine medical check-up plays an essential role in health care. A routine medical check-up can be defined as “a routine health-care process usually done by health-care facilities for both genders and for all age groups at different periods according to the patient risk factors” [16]. Globally, the frequency of routine medical check-ups varies from country to country. In Germany, the percentage of periodic health examinations is 50.8% for men and 49.8% for women [17]. Meanwhile, in Saudi Arabia, only 34.3% of middle-aged and older adults have routine medical check-ups [16]. In Japan, the proportion of people aged 48 and over with routine medical check-ups is 38.4% [18]. Having health insurance, advanced age, high socio-economic characteristics, and excellent social support are factors that facilitate the use of periodical health care services [19]. The benefits of routine medical check-up are undeniable. It can provide information about the health status of service users, detect diseases at an early stage, and helps to plan timely treatment, especially for noncommunicable diseases such as cancer or cardiovascular diseases [20, 21]. Routine medical check-ups help reduces future hospitalization and associated costs as well as improve health and quality of life.

In Vietnam, prior literature indicated that even in urban areas, the proportion of people having routine medical check-ups was only 51.2% [22]. On the other hand, self-treatment among the general population is alarming. A study from 1998 found that the rate of self-treatment in mountainous areas was 57% in the last four weeks [23]. Another recent study showed that 83.3% of people in mountainous areas in Central Vietnam had self-treatment practices in the last 12 months [5]. Self-treatment is mainly based on personal experience or advice from friends, relatives, and drug sellers. With geographic barriers and a shortage of medical services in mountainous areas, it is understandable that people have a high rate of self-treatment when it is difficult to access necessary services in time.

Although benefits of routine medical check-up have been recognized in literature, limited evidence is available that establishes the relationships between routine medical check-ups and self-medication in mountainous people. Previous evidence suggested that one of the most common reasons for self-treatment is the familiarity of or having previous experience with the treatment [24, 25]. Routine medical check-up via individualized counselling can help to increase self-efficacy and self-management [26], which might reduce the likelihood of self-treatment [27]. Thus, we hypothesized that routine medical check-up was associated with a lower likelihood of self-treatment in this population. Understanding this linkage is necessary for further interventions to reduce the self-medication practice in this region. This study was conducted to evaluate the routine medical check-up and self-treatment behavior of people living in a remote and mountainous setting in Vietnam and identify their associations.

## 2. Materials and Methods

### 2.1. Study Design

The study was conducted in August 2018 in Cao Son commune, Da Bac district, Hoa Binh. Cao Son is a typical mountainous commune with rugged terrain, which is divided by many mountains with an average altitude above the sea level of 560 m. The whole locality has nearly 4,000 people with five main ethnic groups including Kinh, Tay, Dao, Muong, and Thai. Criteria for selecting research participants include age 18 and older, who were recognized as having sufficient capability to have their own decision for healthcare seeking. Other inclusion criteria included residing in the local for at least 12 months and agreeing to participate in the study. People who were younger than 18 years of age, did not have sufficient ability to answer the survey (due to illness, alcohol use, or other reasons affecting cognitive ability), and disagree to participate in the study were considered unsuitable for the study.

In this study, we estimated the necessary sample size by using the formula to calculate a population proportion with specified relative precision, with following parameters: confidence level = 95%, expected prevalence = 57% [23], and relative precision = 0.13. The required sample size was 172 residents. The research team will first cooperate with the local authorities and the head of the commune health station to set up a sample frame of people living in the commune based on ethnic characteristics and the age of 18 years or older. Then, we used a simple random sample using a computer software for selecting participants. Those selected were contacted and invited to the commune health centre on a specific day to have a health examination and face-to-face interview. If someone refused, the team would select the next person on the list. The study participants underwent general health examinations which were conducted by physicians from the Vietnam National University Hospital, Hanoi, E Hospital, and St. Paul's Hospital and interviewed by researchers and students from the School of Medicine and Pharmacy, Vietnam National University, Hanoi. There were 180 people who were randomly invited to participate in the study. After data processing, information of 175 people was included in the analysis (97.2% completion rate).

The research team developed a structured questionnaire to use for face-to-face interviews. The content of the questionnaire was built based on the literature review and after considering the context of the study setting. We understood that in the study setting, using standardized scales for measuring self-medication was inappropriate given that the education level of the residents was not high. Therefore, we selected simple questions with detailed instructions. We consulted with local researchers and health workers to optimize contents of the questionnaire. Each interview lasted for 15 minutes. The interviewers were researchers and students of the School of Medicine and Pharmacy, Vietnam National University, Hanoi. These interviewers were trained in communication and interviewing skills. The questionnaire was pretested with five indigenous peoples with different ethnic characteristics before conducting the collection on a larger scale. This pilot study enabled the research team to adjust the content of the questionnaire to the culture and language of the local people.

The questionnaire included following sections: demographic characteristics, health status and health service use and access, and self-treatment. The questionnaire is presented in the Supplemental file (available here).

Demographic information such as age, gender, marital status, ethnicity, occupation, number of people living with, and status of health insurance was collected.

For health status, we asked them to report any acute symptoms and chronic conditions that they suffered in the last four weeks and last three months, respectively. The acute symptoms might include headache, backache, allergy, constipation, cough, sore throat, sneezing/runny nose, fever, worm infections, helminths, diarrhoea, food poisoning, eyesore, or others. Meanwhile, the chronic conditions might include high/low blood pressure, cardiovascular diseases, diabetes, cancer, asthma, epilepsy/psychiatry, HIV AIDS, gastrointestinal disease, osteoarthritis, or other disabilities. People were classified in “Having any acute symptoms in the last four weeks” and “Having any chronic diseases in the last three months” if they reported that they had any of these acute symptoms or chronic conditions. We confirmed the information based on medical examination before the interview. We also asked them to report their health information sources (including friends/relatives; posters/banners; Internet/social media; mobile phone messages; radio, television; loudspeaker; newspapers, books; and health workers).

For measuring health service access, routine medical check-up use, and self-treatment, participants were asked following questions: “What is the nearest medical facility from your home?”, and “How far is the nearest facility from your home?”. Then, we asked them whether they had routine medical check-up in the past 12 months with a question “In the past 12 months, did you have routine medical check-up?” (yes/no) and the frequency of routine medical check-up (“In the past 12 months, how often have you had routine medical check-up?”). We used a series of questions to ask them to report their self-treatment practices. First, we asked the participants to report solutions when they had illness in the past 3 months (“During the past 3 months, when having any symptoms or illnesses, what did you do to handle them?”). If they reported self-treatment practices, they were asked about the symptoms they experienced, kind of medicine they bought, the criteria for medication selection, and the reasons why they bought medicine by themselves.

### 2.2. Statistical Analysis

Data were processed and analyzed using the Stata 15.0 software. Descriptive statistics with percentage and frequency, mean, and standard deviation were calculated. Multivariate logistic regression model was used to assess factors associated with regular medical check-ups as well as the relationship between regular medical check-ups and self-treatment. *p* value <0.05 is used to determine statistical significance.

### 2.3. Ethical Consideration

Research participants were briefly introduced to the study goals and their rights when participating in the study. They have the right to withdraw from the study at any time without affecting the use of health care services at health facilities. They did not receive any compensation for participating in the study. The research proposal was approved by the Institutional Review Board of School of Medicine and Pharmacy, Vietnam National University, Hanoi.

## 3. Results

Among the 175 participants, the average age was 53.2 (SD = 15.7), with the ages ranged from 19 to 90. The majority of respondents were female (76.0%), ethnic minorities (87.8%), and farmers (86.1%). The percentage of people with education from upper secondary and higher was the highest at 46.3%. Most people were married (83.3%) and had health insurance (94.3%). The average number of people living in a household was 4.2 (SD = 1.6) people. The average number of acute symptoms in the past four weeks was 2.9 (SD = 1.6), while the average number of chronic illnesses in the last three months was 1.9 (SD = 1.1) (Table 1).

Table 2 shows that, in the 12 months before the study, 24% of the people had regular medical check-ups; most of them had regular visits every 12 months (70.6%). Most people lived closest to the commune health station (96.0%), with an average distance from home to the nearest medical facility of 4.0 (SD = 4.7) km. The primary source of health information was loudspeakers (51.4%) and radio/television (46.9%). Only 3.4% of participants heard health information from health workers.

Table 3 also shows that the rate of self-treatment in the past three months was 33.7%. The majority of residents had self-treatment when they had headache (63.8%) and fever (32.8%). Pain relief drug and antibiotics were major medicine bought by residents at 36.2%. Most of them reported that they described the symptoms to the pharmacists and bought the drugs followed their recommendations (84.5%). Three most common reasons for self-treatment included “Having previous experience with similar health problems” (29.3%), “Not having time for visiting health facility” (25.9%), and “Mild symptoms” (20.7%).

The regression model results in Table 4 show that the number of chronic diseases (OR = 1.5, 95% CI = 1.0‐2.3), health information sources from radio/television (OR = 3.3, 95% CI = 1.2‐9.5), or social media (OR = 24.8, 95% CI = 1.2‐512.4) was related to routine medical check-up in the past 12 months.

Results of Table 5 show that, after adjusting to other factors, those who did not have regular health checks were six times more likely to self-treat (OR = 6.3, 95% CI = 1.9‐21.1) than those who had a regular medical check in the past 3 months.

## 4. Discussion

This study contributed to the current literature to emphasize a lack of regular medical check-ups, as well as proposed initial relationship between this health behavior and self-treatment. This is particularly important issue in the community, especially in the mountainous settings where self-treatment is very common. Our results showed that a low proportion of people had routine medical check-up in the last 12 months and a moderate prevalence of self-treatment in the last 3 months. Our research also shows the evidence that not having routine medical check-up was closely related to self-treatment among people in this region.

This study shows that the percentage of people with regular health check-ups in the past 12 months was low at 24%. Our finding was significantly lower than the rate in other studies in urban in Vietnam (51.2%) [22] or in the world such as Germany, Saudi Arabia, and Japan [16–18]. Routine medical check-up plays an important role in keeping health service users up-to-date on their health status, as well as screening and detecting chronic conditions that are hidden or at an early stage, which, in turn, helps to make appropriate treatment strategies [19, 22]. However, some studies have shown that the role of routine medical check-up is questioned due to high costs, unclear benefits, or uncertain quality [28–30]. In Vietnam, a previous study showed that patients doubted the quality of doctors and medical services, especially at the primary health care level [31–33]. Our research was conducted in mountainous areas; thus, it was difficult for local people to access health services at higher levels of the health system (such as district or provincial level). Therefore, in order to have routine medical check-up, people could only go to commune health centres, which were the nearest health care facility. According to previous reports, the shortage of human resources and medical equipment at commune health stations in mountainous areas remains serious [34]. As a result, this facility may not be able to provide the necessary medical examination and treatment services for routine medical check-up, making it difficult to motivate people to have regular health check-ups. According to our observations, the available of resources in local commune health stations was consistent with this perception. Although we have not yet assessed the perception of people about the service quality of the commune health station in this study, previous studies have shown that some individuals did not believe in the quality of medical examination and treatment capacity of health staff at the commune health station [31–33]. This could be a major barrier when promoting the routine medical check-up behavior in the mountainous people. This study also found that among those having routine medical check-up, the majority of people had health check-ups at low frequency—once within 12 months, while according to the recommendations of the Ministry of Health, people should have regular health check-ups at least every six months [35]. This phenomenon may be because the economic conditions of the people in this area were low; thus, although most people have health insurance cards, they might still worry that they did not have enough financial resources if they performed regular check-ups more frequently.

Our results were consistent with some previous studies showing that people who suffered from diseases or illnesses were more likely than other people to have regular health check-ups [22]. This result may be explained by the fact that when they have regular health check-ups, their diseases could be detected early as well as treated and managed on time. On the other hand, our research emphasized the importance of some health communication channels such as radio/television or social media in promoting people to get regular medical check-ups. Indeed, the main sources of health information for local people were radio/television, friends/relatives, and loudspeakers. Meanwhile, the role of local health workers in health communication and education in the community had not been emphasized. We assumed that, even if people were communicated about the benefits of regular medical check-ups via these common channels, their awareness of this issue may be inadequate, thereby reducing their belief in this health behavior. Research in Japan shows that belief in routine medical check-up was an important factor in promoting this service use [36]. Therefore, designing cultural communication messages of local people and taking advantage of the popularity of these communication channels to increase awareness and health belief to promote health behaviors and routine medical check-up are needed.

In this study, we found that 33.7% of people self-treated during the past three months. This result was lower than in some studies in the world and Vietnam [5, 23, 37–40]. This difference is probably due to the different recall times, as previous studies often asked for 12 months, while our study asked for three months because we want to limit recall bias which occur when people have to remember events for long periods. Moreover, other characteristics such as sociodemographic characteristics or healthcare accessibility should also be considered. For example, in our setting, there were less than five drug stores in the local community, and none of private clinic was found, suggesting that people in this locality has limited access to health care as well as drug sellers. Results of our study were similar to some previous studies when they showed that in people with self-treatment behaviors, they frequently went to pharmacy stores and consulted with pharmacists to buy drugs as a main information source for decision-making [5, 41]. It is important to consider that more than one-third of self-medicating people buy antibiotics without a prescription. Vietnam has been well-documented as a hotspot of antibiotic resistance [42]. A prior study among mountainous residents in Vietnam found limited knowledge and awareness of these people about antibiotic resistance and appropriate antibiotic use [43]. Our finding suggested the need to design effective strategies to manage self-treatment behavior in the mountainous area.

This study confirmed our hypothesis when showing that routine medical check-up was negatively associated with self-treatment. Indeed, people who had routine medical check-up could be consulted about the current disease, as well as how to handle common symptoms, thereby improving their self-management capacity, as well as their knowledge and attitude toward self-treatment, which in turn reduced the self-treatment practices [26, 27]. On the other hand, people who had regular health check-ups might have more relationships with doctors so that they can consult about treatment at all times, which can reduce their self-treatment behavior.

This study has some limitations. First, the study has not yet analyzed factors that were barriers or facilitators to promote routine medical check-ups. A qualitative study is needed to investigate reasons for using regular medical check-up service. Secondly, the study was conducted in a mountainous area of Northern Vietnam; thus, the results might not reflect the situation in other regions. Third, the study used a cross-sectional research design; hence, it was not possible to conclude a causal relationship between the factors. Finally, the study collected self-reported data, which may lead to the recall bias.

## 5. Conclusions

This study emphasized a low rate of routine medical check-up among residents in mountainous areas in Northern Vietnam. Radio/television and social media as main information sources were associated with routine medical check-up. We also found a high rate of self-treatment in the sample and the negative associations between routine medical check-up and self-treatment practices. Promoting health education and communication through mass media to raise people's awareness about regular health check-ups is a promising way to improve people's self-treatment status.

## Figures and Tables

**Table 1:** Sociodemographic characteristics and health status.

Characteristics	*n*	%
Gender		
Male	42	24.0
Female	133	76.0
Ethnic		
Kinh	21	12.2
Tay	62	36.1
Dao	40	23.3
Muong	49	28.5
Job		
Farmer	148	86.1
Others	24	14.0
Education		
Elementary	15	8.6
Secondary	79	45.1
High school and above	81	46.3
Marital status		
Single	29	16.7
Married	145	83.3
Health insurance		
Yes	164	94.3
No	10	5.8
Having acute symptoms in the last 4 weeks		
Yes	171	97.7
No	4	2.3
Having chronic diseases in the last 3 months		
Yes	162	92.6
No	13	7.4
	Mean	SD
Age	53.2	15.7
Number of people living in a household	4.2	1.6
Number of acute diseases	2.9	1.6
Number of chronic diseases	1.9	1.1

**Table 2:** Healthcare access and routine medical check-up.

Characteristics	*n*	%
The nearest medical facility (*n* = 175)		
Commune health stations	168	96.0
Others	7	4.0
Routine medical check-up in the last 12 months (*n* = 175)		
Yes	42	24.0
No	133	76.0
Frequency of routine medical check-up in the last 12 months (*n* = 42)		
Every three months	25	59.5
Every six months	7	16.7
Every 12 months	7	16.7
≥12 months	3	7.1
Health information sources (*n* = 175)		
Friends/relatives	66	37.7
Television, radio	82	46.9
Loudspeaker	90	51.4
Health worker	6	3.4
Social media	3	1.7
Distance to the nearest facility (km), mean (SD)	4.0 (4.7)	

**Table 3:** Self-treatment practice.

Characteristics	*n*	%
Self-treatment practice in the last three months (*n* = 175)		
No	114	66.3
Yes	58	33.7
Symptoms when practicing self-treatment (*n* = 58)		
Fever	19	32.8
Headache	37	63.8
Stomachache	9	15.5
Cough	12	20.7
Osteoarthritis pain	12	20.7
Allergy	2	3.4
Other	10	17.2
Type of medicine bought (*n* = 58)		
Pain relief	21	36.2
Antibiotics	21	36.2
Others	16	27.6
Criteria when buying medicine for self-treatment (*n* = 58)		
Remember drug's name	3	5.2
Using prescriptions in previous medical examination	3	5.2
Describing symptoms to pharmacists	49	84.5
Using drugs available at home	2	3.4
Others	1	1.7
Reasons for self-treatment (*n* = 58)		
Having previous experience with similar health problems	17	29.3
Mild symptoms	12	20.7
Not having time for visiting health facility	15	25.9
Not having enough money for visiting health facility	5	8.6
Medical facility is far away	8	13.8
Confidentiality	1	1.7

**Table 4:** Associated factors with a routine medical check-up.

Factors	Routine medical check-up			
	OR	*p*	95% CI	
Ethnic				
Kinh	Ref			
Tay	0.6	0.43	0.1	2.2
Dao	0.2	0.07	0.0	1.2
Muong	0.8	0.80	0.2	3.2
Gender				
Male	Ref			
Female	0.7	0.46	0.3	1.8
Job				
Famer	Ref			
Others	0.4	0.26	0.1	1.8
Education				
Elementary	Ref			
Secondary	0.3	0.14	0.1	1.5
High school and above	0.2	0.08	0.0	1.2
Health insurance				
No	Ref			
Yes	2.9	0.21	0.6	14.7
Receiving medical information from relatives/friends				
No	Ref			
Yes	2.0	0.23	0.6	6.0
Receiving medical information from television, radio				
No	Ref			
Yes	3.3	0.02	1.2	9.5
Receiving medical information from the loudspeaker				
No	Ref			
Yes	0.4	0.09	0.1	1.2
Receiving medical information from a medical worker				
No	Ref			
Yes	0.7	0.78	0.1	8.1
Receiving medical information from social media				
No	Ref			
Yes	24.8	0.04	1.2	512.4
Number of people living in a household	0.9	0.64	0.7	1.2
Number of acute diseases	1.0	0.74	0.7	1.3
Number of chronic diseases	1.5	0.03	1.0	2.3
Distance to the nearest facility (km)	1.1	0.22	1.0	1.1

**Table 5:** Association between self-treatment and routine medical check-up.

Factors	Self-treatment			
	OR	*p* value	95% CI	
Routine medical check-up				
Yes	Ref			
No	6.3	0.00	1.9	21.1
Ethnic				
Kinh	Ref			
Tay	0.5	0.32	0.2	1.8
Dao	1.3	0.73	0.3	5.9
Muong	0.5	0.34	0.1	1.9
Gender				
Male	Ref			
Female	1.5	0.37	0.6	3.8
Job				
Famer	Ref			
Others	1.0	0.96	0.3	3.7
Education				
Elementary	Ref			
Secondary	2.1	0.37	0.4	10.9
High school and above	2.1	0.40	0.4	12.3
Health insurance				
No	Ref			
Yes	0.8	0.76	0.1	4.7
Receiving medical information from an acquaintance				
No	Ref			
Yes	0.5	0.13	0.2	1.3
Receiving medical information from television/radio				
No	Ref			
Yes	1.1	0.91	0.4	2.6
Receiving medical information from the loudspeaker				
No	Ref			
Yes	0.7	0.46	0.3	1.8
Receiving medical information from a medical worker				
No	Ref			
Yes	0.3	0.40	0.0	4.1
Receiving medical information from media network				
No	Ref			
Yes	2.5	0.55	0.1	46.2
Number of people living in a household	1.2	0.13	0.9	1.5
Number of acute diseases	1.1	0.68	0.8	1.3
Number of chronic diseases	1.0	0.83	0.7	1.4
Distance to the nearest facility (km)	1.0	0.74	0.9	1.1

## Data Availability

A data availability statement is compulsory for research articles and clinical trials. Here, requests for access to individual subject data may be made to Tuan A. Le; please send an email to tuanla.smp@gmail.com/anhtuan048@gmail.com.

## Supplementary Materials

Supplementary Materials Supplemental file 1: questionnaire of the study.
