# Supplementary material for: Routine Medical Check-Up and Self-Treatment Practices among Community-Dwelling Living in a Mountainous Area of Northern Vietnam
Source: Biomed Res Int. 2021 Apr 22;2021:8734615. doi: 10.1155/2021/8734615 (PMC8087461; doi:10.1155/2021/8734615)
Supplement: Supplementary Materials — Supplemental file 1: questionnaire of the study. [file 8734615.f1.doc]

QUESTIONNAIRE

A PERSONAL INFORMATION

1. Date of Birth: ……………………

2. Gender

1. Male
2. Female

3. What level of education have you completed?

1. Illiterate
2. Primary School
3. Secondary school
4. High school
5. Intermediate/ colleges/ vocational school
6. University
7. Postgraduate

4. What is your current marital status?

1. Single
2. Live with wife / husband
3. Living together as husband / wife, unmarried
4. Divorced / separated
5. Widowed

5. Which ethnic group are you?

1. Kinh
2. Tày
3. Dao
4. Thái
5. Other…………………

6. What is your occupation?

1. Retirement
2. Unemployment
3. Farmer
4. Worker
5. Administrative staff
6. Student
7. Other:………………………………………………

7. What is your family's average monthly gross income? ………………. (VND)

8. How many people in your family have eaten together in the past 3 months? ……………….. people

9. Do you have health insurance?

1. Yes
2. No

B. HEALTH INFORMATION

1. In the past 4 weeks, have you had any of the following symptoms? (Multiple choices)

1. Headache
2. Backache
3. Allergy
4. Constipation
5. Cough, sore throat
6. Sneezing / runny nose
7. Fever
8. Worm infections, helminths
9. Diarrhea
10. Gynecological disease
11. Skin diseases
12. Food poisoning
13. Eyesore
14. Other: ……………………

2. In the past 3 months, have you had the following chronic illnesses? (Multiple choices)

1. High blood pressure
2. Heart-related disease
3. Diabetes
4. Cancer
5. Asthma
6. Polio / Paralysis
7. Epilepsy / Psychiatry
8. HIV AIDS
9. Stomach disease / Gastrointestinal disease
10. Deaf
11. Spine pain / osteoarthritis
12. Low blood pressure
13. Other: ……………………………………..

C. HEALTH SERVICES USE

1. What is the nearest medical facility from your home?

1. Central hospital
2. Provincial Hospital /Medical Center
3. District Hospital /Medical Center
4. Commune health station
5. Private medical facility
6. Other:……………………………..

2. How far is the nearest facility from your home? …………… km

3. During the past 3 months, when having any symptoms or illnesses, what did you do to handle them

1. Not sick
2. Go to the medical facility to see a doctor
3. Self-medication / Self-medication
4. Wait self out
5. To the healer's house
6. Worship
7. Other:………………………………..

4. If self-medication, at that time, what illness or symptoms did you have?

1. Fever
2. Headache
3. Stomachache
4. Cough
5. Arthritis
6. Allergy
7. Other:………………………………

5. If you bought medicine by yourself, what criteria did you base on buying medicine?

1. Remember drug’s name
2. Using prescriptions in previous medical examination
3. Describing symptoms to pharmacists
4. Using drugs available at home
5. Others

6 .Why did you buy medicine yourself?

1. Having previous experience with similar health problems
2. Mild symptoms
3. Not having time for visiting health facility
4. Not having enough money for visiting health facility
5. Medical facility is far away
6. Confidentiality
7. Other:……………………………………..

7. What kind of medicine did you buy?

1. Pain relief
2. Antibiotics
3. Other:……………………………………….

8. In the past 12 months, have you had regular checkups?

1. Yes
2. No

9. In the past 12 months, how often have you had regular checkups?

1. 3 months
2. 6 months
3. 12 months
4. 12 months
5. Other…………………

10. What are your health information sources? (Multiple choice)

1. Friends / relatives
2. Posters / banners
3. Internet
4. Phone messages
5. Radio, television
6. Loudspeaker
7. Newspapers, books
8. Medical staff
9. Social Network
10. Others ………………………………………… ..
